# Selective Antineoplastic Potential of Fractionated Caribbean Native *Ganoderma* Species Extracts on Triple-Negative Breast Cancer Cells

**DOI:** 10.3390/ph17070864

**Published:** 2024-07-01

**Authors:** Luz V. Arroyo-Cruz, Sebastián Sagardía-González, Kurt Miller, Taotao Ling, Fatima Rivas, Michelle M. Martínez-Montemayor

**Affiliations:** 1 Department of Biochemistry, School of Medicine, Universidad Central del Caribe, Bayamón 00960-6032, Puerto Rico; luz.arroyo@uccaribe.edu; 2 Huerto Rico, Carolina 00987, Puerto Rico; sebastian@huertoricopr.com (S.S.-G.); kurt@huertoricopr.com (K.M.); 3 Department of Chemistry, Louisiana State University, 133 Choppin Hall, Baton Rouge, LA 70803, USA; tling@lsu.edu (T.L.); frivas@lsu.edu (F.R.)

**Keywords:** triple-negative breast cancer, natural product, *Ganoderma multiplicatum*, *Ganoderma martinicense*, fractions, cytotoxicity, SUM149PT, MCF-10A

## Abstract

Triple-negative breast cancer (TNBC) is an aggressive subtype characterized by the absence of estrogen receptor, progesterone receptor, and human epidermal growth factor receptor type 2 expression. It is known for its high malignancy, invasiveness, and propensity for metastasis, resulting in a poor prognosis due to the absence of beneficial therapeutic targets. Natural products derived from mushrooms have gained significant attention in neoplastic therapy due to their potential medicinal properties. The therapeutic potential of *Ganoderma lucidum* in breast cancer has been highlighted by our group, suggesting its use as an adjuvant treatment. The present study aims to assess the potential antineoplastic capacity of two Caribbean native *Ganoderma* species found in Puerto Rico, *Ganoderma multiplicatum* (*G. multiplicatum*) and *Ganoderma martinicense* (*G. martinicense*). Antiproliferative studies were conducted via cell viability assays after cultivation, harvesting, and fractionation of both species. The obtained results indicate that most of the fractions show some cytotoxicity against all cell lines, but 33% of the fractions (F1, F2, F7, F12) display selectivity towards cancer cell models. We demonstrate for the first time that native *Ganoderma* species can generate metabolites with anti-TNBC properties. Future avenues will focus on structure elucidation of the most active fractions of these *Ganoderma* extracts.

## 1. Introduction

Cancer, in its various manifestations, is a battle that continues to challenge humanity. Among its most elusive variants is triple-negative breast cancer (TNBC), a disease that demands new strategies in the fight for life. Globally, TNBC accounts for about 15–20% of all breast cancer (BC) cases [1,2]. Between 8.8 and 15% of BC cases in the United States are TNBC, which is more common in African American and Hispanic women [3,4,5]. In Puerto Rico, TNBC tumors account for 9.5% of BC cases [6]. The five-year survival rate for TNBC is 77%, which is significantly lower than those for other BC subtypes (93%), underscoring the urgent need for more effective therapies [4,7,8]. TNBC is a subtype characterized by the absence of three specific markers in cancer cells: estrogen receptors (ERs), progesterone receptors (PRs), and human epidermal growth factor receptor type 2 (HER2) [7]. Being negative for ERs, PRs, and HER2 makes TNBC particularly challenging to treat, as it does not respond to targeted therapies that are effective in BC tumors that display these receptors [1,9]. Finally, TNBC tends to be more aggressive and metastasizes quicker than other BC types, and is often diagnosed in advanced stages, further complicating treatment [10].

In the search for innovative solutions against TNBC, we explored the promising properties of extracts from the medicinal mushroom *Ganoderma* species. *Ganoderma* genus, commonly known as “Reishi” in Japan or “Lingzhi” in China, have been used for centuries in traditional Chinese and Japanese medicine for their health benefits [11,12]. *Ganoderma* species contain a variety of bioactive compounds, such as triterpenoids and polysaccharides, which have been studied for their potential medicinal qualities [13,14,15,16,17,18,19,20,21]. Previously, we reported the properties of ergosterol peroxide (EP), a bioactive compound isolated from the *Ganoderma lucidum* (*G. lucidum*) mushroom. We show EP’s selective anti-cancer properties in a TNBC model [22,23,24,25,26].

Different fractions extracted from *Ganoderma* spp. exhibit varying levels of cytotoxicity against triple-negative breast cancer (TNBC) cells due to their diverse bioactive compounds, including lanostane triterpenoids, meroterpenoid dimers, triterpenes, and polysaccharides [13,27,28,29,30]. These compounds target different pathways, including cancer cell growth and survival, apoptosis, cell proliferation, and modulating oxidative stress pathways, resulting in differential cytotoxic effects.

Certainly, it is widely recognized that environmental factors play a fundamental role in mushroom morphology, shape, size, and context color, and the production of secondary metabolites [31,32,33]. The stress to which wild species are subjected can act as the trigger for the production and concentration of these metabolites [32,33,34]. In the Caribbean Island of Puerto Rico, neotropical species of *Ganoderma* can be found in the wild that are morphologically similar to and allied with *G. lucidum*.

In this brief report, we explore an exceptional natural resource, neotropical extracts from *Ganoderma multiplicatum* (*G. multiplicatum*) and *Ganoderma martinicense* (*G. martinicense*), in the search for selective therapeutic alternatives against TNBC. We hypothesize that these Puerto Rican native *Ganoderma* spp. (*G. multiplicatum* and *G. martinicense*) are potential producers of anti-TNBC compounds. To investigate whether native *Ganoderma* species (*G. multiplicatum* and *G. martinicense*) had selective cytotoxicity against TNBC cells, large-scale cultures of both native *G. multiplicatum* and *G. martinicense* were conducted, followed by alcoholic extraction and fractionation of their individual extracts. Cell viability assays were performed to determine the anti-cancer potential of each fraction that was divided based on polarity and mass [35]. Our work demonstrates for the first time the selective antineoplastic potential of native Puerto Rican *Ganoderma* species on TNBC cells, while showing no detectable cytotoxicity to noncancerous cell models.

## 2. Materials and Methods

### 2.1. Experimental Native Ganoderma spp. Fungi Procedures

#### 2.1.1. Collection and Isolation

**Figure 1:**
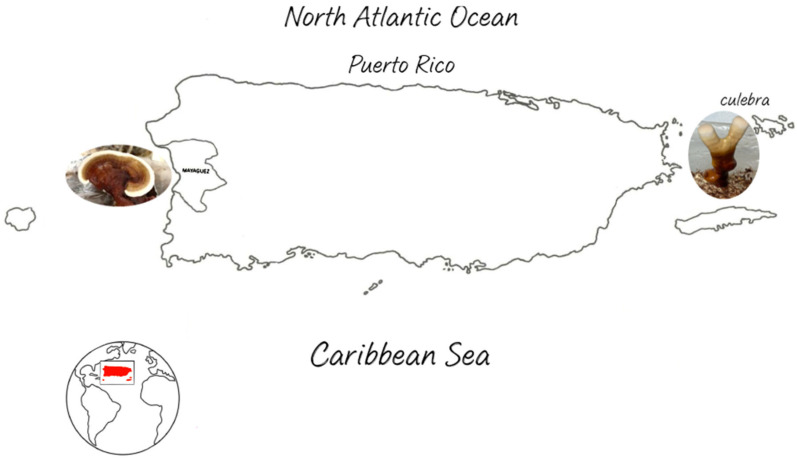
Locations in Puerto Rico where the *Ganoderma* species were collected. Wild samples of the *Ganoderma* spp. were harvested from logs, tree stumps, and otherwise declining trees in different habitats across Puerto Rico. *G. multiplicatum* was collected in a forest in Miradero, Mayagüez, and *G. martinicense* was collected near Flamenco Beach, Culebra. (Map author: Mireliz A. Arroyo-Cruz).

Wild samples of the *Ganoderma* complex (*Ganoderma* sect. *Ganoderma*, those with “varnished” or laccate pilei) were harvested from logs, tree stumps, and otherwise declining trees in different habitats across Puerto Rico (Figure 1). *G. multiplicatum* was collected in June (Mushroom Observer #: MO 369977; Cultural Code: PR30; collection date: 18 June 2019; GPS: 18.1314, −67.1386) and July (Mushroom Observer #: MO 373519; collection date: 9 July 2019; GPS: 18.1314, −67.1192) 2019 in a forest in Miradero, Mayagüez, Puerto Rico. *G. martinicense* was collected in September 2019 (Mushroom Observer #: MO 383819; collection date: 23 September 2019; GPS: 18.32865, −65.316) near Flamenco beach, Culebra, Puerto Rico. Fruiting bodies were photographed in situ and sent to the Department of Plant Pathology, University of Minnesota, for molecular identification. Mushroom tissue was harvested from the context of the fresh collections within a week of harvest and transferred to a selective medium for Basidiomycota according to Loyd et al.’s specifications [34]. Once the medium was colonized, the mycelium was isolated and transferred to malt extract agar (MEA) without antibiotic or other supplements.

#### 2.1.2. Identification

Sequences of the genetic markers were obtained from pure *Ganoderma* cultures on MEA. The DNA was extracted, amplified, and sequenced at the University of Minnesota using previously described methods [36]. Cultures were stored on agar slants, and dried *Ganoderma* fruiting bodies were placed in frozen storage pending ongoing taxonomic work.

#### 2.1.3. Culture and Harvest

MEA cultures of the species *G. multiplicatum* (Supplementary Materials, Figure S1a), and *G. martinicense* (Supplementary Materials, Figure S1b) were mailed to the Huerto Rico, LLC laboratory, which was in Bayamón, Puerto Rico, to test the viability of commercial cultivation. Agar cultures were transferred to a sterilized rye grain substrate, prepared by mixing rye grains with 1% gypsum (CaSO_4_ 2H_2_O) in 16 oz wide-mouth mason jars with filtered lids and hydrating them to 55% ± 3% and sterilizing them at 15 psi (120 °C) for one hour. Once cooled, the grain spawn was inoculated by aseptically placing squares of colonized MEA into bags of sterilized grain spawn in front of a flow hood and agitating the bags to distribute the inoculant. Bags were then sealed and placed in a dark incubation room at room temperature until fully colonized.

The colonized grain spawn was used to further inoculate a bulk substrate consisting of a 1:1 mix of hardwood sawdust and woodchips from a local wood mill. The source of the wood was local angiosperm trees, including cahoba (*Swietenia* spp.), almendrón (*Terminalia catappa*), and majó (*Hibiscus elatus*). Once combined, the woodchips and sawdust were mixed with gypsum (CaSO_4_ 2H_2_O) at a rate of 2%, then hydrated to 65% ± 3% water content and loaded into polypropylene autoclave bags (8.25 × 4.75 in). Bags were sterilized at 15 psi (120 °C) for one hour in an autoclave. The cooled bulk spawn was inoculated with the colonized grain spawn in front of a flow hood at a ratio of 1:10, agitated, and allowed to fully colonize the substrate in a dark room at 27 °C.

The fully colonized spawn bags were cut open to allow oxygen exchange and stimulate growth, then moved to a climate-controlled fruiting room, and maintained at 90% humidity and 21 °C for two months while the *Ganoderma* fruiting bodies developed. The fruiting bodies were harvested at the beginning of spore production when the fruiting bodies had a shelf-like appearance.

#### 2.1.4. Drying and Pulverization

Harvested fruit bodies of the cultivated *Ganoderma* species were placed in a food dehydrator or sliced into smaller units to fit the dehydrator shelves. The material was dehydrated for 24–48 h at 43–42 °C or until moisture was evaporated and the fruiting bodies were rendered brittle and lightweight. This material was further snapped by hand or cut with a knife until small enough to mill through a Baratza Encore burr grinder on a fine grind setting. The resulting powder was stored in an airtight Ziploc bag (Supplementary Materials, Figure S1c).

### 2.2. Native Ganoderma spp. Experimental Chemistry Procedures

#### Fractionation

The dried, pulverized specimen stem and mushroom body (100 g) were placed in a Soxhlet extractor setup and extracted with 600 mL of isopropanol under re-fluxing conditions for 48 h. The resulting mixture was filtered and concentrated under vacuum at 35 °C using a rotary evaporator (Buchi 215, Buchi Corporation, New Castle, DE, USA), which afforded a dark brown gum (9.5 g). Due to the experimental scale limitation, the remaining mushroom tissue was exposed to another 48 h extraction with CH_2_Cl_2_ and later combined with the initial mixture to provide an additional 0.7 g of the extract. The mixture was subjected to silica gel chromatographic separation (5.8 × 16 cm, EtOAc−nHexane, step gradient elution 0:100, 20:80, 40:60, 60:40, 80:20, and 100:0, and then with acetone) to obtain 13 fractions. LC-MS and TLC analyses of the fractions derived from these partition processes revealed that the fractions contained major nonpolar compounds with characteristic colors (Supplementary Materials, Figure S2) indicating terpenes when sprayed with anisaldehyde sulfuric acid after TLC analysis.

### 2.3. Experimental Breast Cancer Cellular Procedure

#### 2.3.1. Cell Culture

The human TNBC cell line SUM149PT (CVCL_3422) was obtained from BioIVT LLC (Westbury, NY, USA) and cultured in Ham’s F-12 Nutrient Mix (Life Technologies, Carlsbad, CA, USA) supplemented with 10% or 5% fetal bovine serum (FBS; Corning, NY, USA) as in [24]. The human noncancerous mammary epithelial cell line MCF-10A (CVCL_0598, ATCC^®^ CRL-10317™) was obtained from American Type Culture Collection (ATCC^®^, Manassas, VA, USA) and was cultured in DMEM/Ham’s F12 (Life Technologies, Carlsbad, CA, USA) with 10% or 5% horse serum (HR; Sigma-Aldrich, Inc., St. Louis, MO, USA) as described in [22]. Culture media components were purchased from Life Technologies/Gibco (Rockville, MD, USA) [22]. All cell lines were incubated at 37 °C and maintained in an atmosphere containing 5% CO_2_ in accordance with sterile cell culture practices [37]. Cells were tested regularly to ensure they were free from mycoplasma infection using the *Mycoplasma* Detection Kit (ASB-1310001, Nordic BioSite AB, Täby, Sweden).

#### 2.3.2. Cell Viability Assay

Thirty thousand cells/well of the SUM149PT cell line or fifty thousand cells/well of the MCF-10A cells were seeded in 48-well plates in 10% FBS or HS media and cultured for 24 h. Then, the media cells were changed to 5% FBS or HS, continuing with 1 h of incubation. Next, cells were treated in duplicate with the vehicle (0.13%, 0.75% or 0.50% DMSO) or each of the *G. multiplicatum* and *G. martinicense* fractions (0–12.5 µM, 0–75 µM or 0–50 µM, concentration curve) for 72 h of incubation. After the treatment period, the cells were fixed (cold methanol) and the nuclei were stained [0.4% propidium iodide (PI) (Sigma Aldrich, Inc., St. Louis, MO, USA)]. Fluorescence units were measured using a GloMax^®^ Microplate Reader (Promega, Madison, WI, USA). Cell viability was calculated as the percent of surviving cells after treatment relative to the vehicle as in [24].

### 2.4. Data Analysis

We expressed data as mean ± S.E.M., and calculated *p*-values using nonparametric *t*-tests with the Mann–Whitney test. We performed statistical analyses using Graph Pad Prism v. 10.01 (San Diego, CA, USA), and we considered differences significant when *p* ≤ 0.05. We calculated the half-maximal inhibitory concentration (IC_50_) using dose–response curve fittings with the nonlinear regression parameter dose–response–inhibition using Graph Pad Prism. We conducted experiments in three or more independent biological replicates and calculated the mean by adding the independent replicates of each experiment and dividing the value by the total number of independent replicates (e.g., *n* = 3).

## 3. Results

### 3.1. Caribbean Native Ganoderma spp.

Two native *Ganoderma* species (*G. multiplicatum* and *G. martinicense*) were collected, isolated, identified, and cultivated to obtain the harvest shown in Supplementary Figure S1a,b, respectively. After the drying and spraying steps (Supplementary Materials, Figure S1c), the samples were sent to Louisiana State University for chemical processing. Fractionation was conducted on the native *Ganoderma* species to obtain seven fractions from *G. multiplicatum* and six fractions from *G. martinicense* (Supplementary Materials, Figure S2).

### 3.2. Antineoplastic Potential of Native Ganoderma spp. on TNBC Cells

To determine the cytotoxicity of each fraction on TNBC (SUM149PT) or noncancerous cells (MCF-10A), cell viability assays were performed using each of the 13 fractions from *G. multiplicatum* and *G. martinicense*. Table 1 shows the results of the mean inhibitory concentrations (IC_50_) and statistically significant differences between cells (*p*-value) of the medicinal mushroom fractions evaluated. *G. martinicense* F14 did not cause any toxicity on either the cancerous or the noncancerous cells evaluated. Therefore, its values are not provided on the table below.

**Figure 2:**
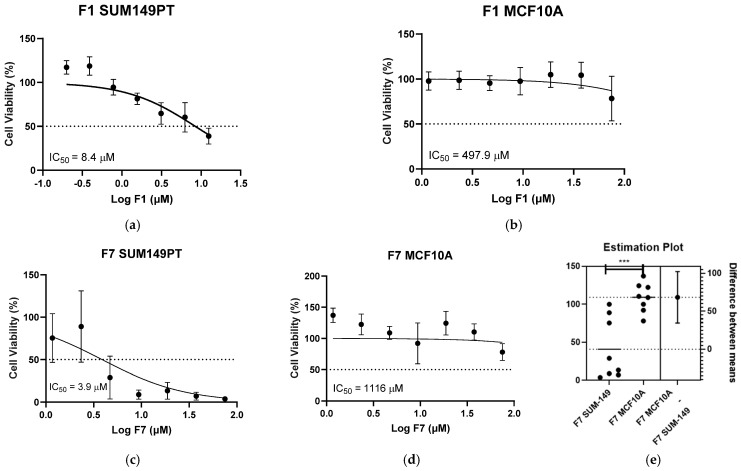
Effects of Caribbean native *G. multiplicatum* medicinal fractions in TNBC (SUM149PT) and noncancerous (MCF-10A) cell lines. SUM149PT and MCF-10A cells were seeded and treated. (**a**,**b**) Cytotoxic effects of compound F1 against SUM149PT and MCF-10A, respectively. The TI for F1 resulted in a value of 59.3. (**c**,**d**) Great cytotoxic effects of compound F7 against SUM149PT and MCF-10A, respectively. The TI for F7 resulted in a value of 286.2. (**e**) Significant difference (*p*-value) of compound F7 determined between SUM149PT and MCF-10A cell means. Bars represent mean ± SEM of at least 3 biological replicates. *** *p* < 0.0001 when comparing SUM149PT and MCF-10A cell means.

Twelve fractions were evaluated, and those that exhibited the greatest bioactivity against TNBC SUM149PT cells were F7 (IC_50_ = 3.9 µM, *p <* 0.0007) and F1 (IC_50_ = 8.4 µM) (Figure 2). These effects occurred exclusively on cancer cells, without having significant adverse bioactivity on noncancerous MCF-10A cells (IC_50_ = 1116 µM, and IC_50_ = 497.9 µM, respectively), providing therapeutic indices (TIs) of 286.2 and 59.3, respectively. Fractions F2 (IC_50_ = 11.3 µM) and F12 (IC_50_ = 11.5 µM) closely followed in bioactivity potency against SUM149PT cells (Supplementary Materials, Figures S3–S6). Our findings show for the first time the efficient antineoplastic potential and selectivity of fractions from native Puerto Rican *Ganoderma* species on TNBC cells. Interestingly, of all the fractions evaluated, only F6 (IC_50_ = 15.4 µM, *p* < 0.05) exhibited greater bioactivity against MCF-10A cells (Supplementary Materials, Figures S3–S6) compared to SUM149PT cancer cells.

## 4. Discussion

TNBC presents a formidable challenge in oncology due to its aggressive nature and limited treatment options, highlighting the urgent need for innovative therapeutic strategies [38,39]. Our study elucidates the potential of natural products, specifically medicinal mushrooms of the *Ganoderma* genus, as sources of anti-TNBC compounds. TNBC, characterized by the absence of ERs, PRs, and HER2 expression, is notorious for its high malignancy, invasiveness, and propensity for metastasis, necessitating multimodal chemotherapy with limited efficacy [40,41].

Phytochemicals derived from plants have shown significant potential in breast cancer therapy by targeting various pathways involved in cancer progression. They can reduce cell proliferation, induce apoptosis, decrease metastasis, suppress angiogenesis, and reduce the migratory properties of cancer cells [42,43]. These compounds affect both estrogen-dependent and estrogen-independent breast cancer cell proliferation and breast cancer stem cells [44]. Research on phytochemicals like Indol-3-Carbinol, Resveratrol, Curcumin, Quercetin, and others has demonstrated promising results in breast cancer cell lines [45,46]. Dietary recommendations for breast cancer patients emphasize consuming foods rich in flavonol polyphenols to potentially reduce the cancer recurrence risk [47]. Additionally, phytochemicals are being explored as adjuvants to conventional treatments due to their antioxidant properties [48].

**Figure 3:**
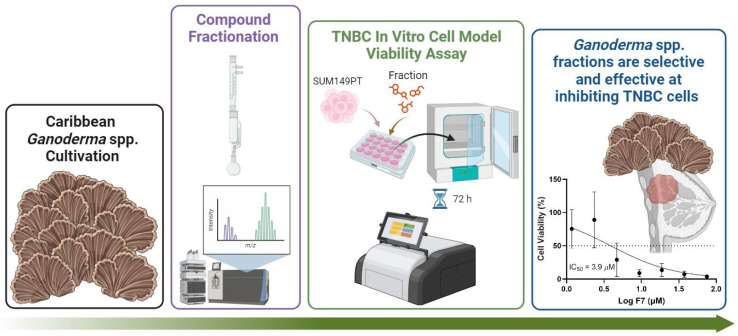
Graphical summary of this study. We assessed the antineoplastic capacity of two Caribbean native species of *Ganoderma* found in Puerto Rico: *Ganoderma multiplicatum* (collected at a forest in Miradero, Mayagüez) and *Ganoderma martinicense* (collected near Flamenco Beach, Culebra). We conducted a cell viability assay after cultivating, harvesting, and fractionating both species. The obtained results indicate that most of the fractions show some cytotoxicity against all cell lines, but 33% of the fractions (F1, F2, F7, F12) display selectivity towards cancer cell models. This summary figure was created using BioRender.com.

The exploration of medicinal mushrooms in cancer therapy has garnered significant interest, emphasizing the importance of integrative approaches in oncology [49,50]. *G. lucidum*, renowned for its immunomodulatory and antineoplastic properties, has been extensively studied [22,23,24,25,29,51,52,53,54]. Our investigation extends this research by evaluating the anti-TNBC potential of two understudied, native *Ganoderma* species found in Puerto Rico (Figure 3), *G. multiplicatum*, and *G. martinicense*.

It should be noted that these species of *Ganoderma* are not exclusive to Puerto Rico. *G. multiplicatum* is a pantropical species originally described in Venezuela, but it has also been reported in Brazil, French Guyana, China, New Guinea, and Egypt, and recently in Iran, Vietnam, and India [55,56,57,58]. *G. martinicense* is a neotropical species distributed from the Caribbean area to Uruguay and Argentina but is also a new record for North America [32,33,57,59].

Our findings reveal that most fractions from both *Ganoderma* species exhibited selective inhibition of TNBC cells, underscoring their potential as sources of anti-TNBC compounds. Particularly noteworthy is the observation that fractions F1, F2, and F7 from *G. multiplicatum* and F12 from *G. martinicense* displayed high cytotoxicity against TNBC cells while sparing noncancerous cells, suggesting a promising avenue for further exploration. Only fraction F6 from *G. multiplicatum* presented a high cytotoxic capacity against noncancerous cells. In general, *G. multiplicatum* exhibited greater antineoplastic potential compared to *G. martinicense*, with a limited amount of literature documenting the latter’s properties. Researchers only reported that *G. multiplicatum* showed good antibacterial and antifungal activity [13,58]. At the time of this study, to the best of our knowledge, no other literature has been found that documents any properties of *G. martinicense*.

Our study fills a crucial gap by demonstrating the anti-TNBC activity of Caribbean native *Ganoderma* species, highlighting the importance of environmental factors in secondary metabolite production [32,34]. Moving forward, it is imperative to identify and characterize the active compounds present in the most promising fractions, paving the way for in-depth mechanistic studies and preclinical evaluations. Furthermore, elucidating the mechanisms of action and potential clinical applications of *Ganoderma* species in TNBC treatment warrants further investigation.

Using *G. martinicense* and *G. multiplicatum* as therapeutics may be an effective strategy for the chemoprevention and adjuvant treatment of triple-negative breast cancer, highlighting the need for additional research to characterize their compounds and evaluate their clinical impact. Among our limitations, we must highlight the current absence of a phytochemical analysis. These are steps that we will be completing with the progress of a later investigation.

In conclusion, our study underscores the therapeutic potential of Caribbean native *Ganoderma* species as sources of anti-TNBC compounds [14]. Future research endeavors should focus on harnessing the bioactive components of *Ganoderma* for the development of targeted therapies against TNBC, ultimately improving clinical outcomes for patients with this aggressive subtype of breast cancer.

## Figures and Tables

**Table 1:** Cytotoxicity of two Caribbean native *Ganoderma* species extracts, determined via the cell viability assay.

Medicinal Mushroom	Fraction No.	IC_50_ (µM)		Statistically Significant Differences (*p*-Value)	Therapeutic Index (TI) (MCF-10A/SUM149PT)
		SUM149PT	MCF-10A		
*Ganoderma multiplicatum*	1	8.4	497.9	NDM ^1^	59.3
	2	11.3	>75	NDM ^1^	>6.6
	3	40.4	>75	0.0021	>1.9
	4	49.2	>75	0.0110	>1.5
	5	198.4	>75	0.0009	>0.4
	6	110	15.4	0.0461	0.1
	7	3.9	1116	0.0007	286.2
*Ganoderma martinicense*	11	68.7	4.9 × 10^9^	0.0084	7.2 × 10^11^
	12	11.5	>50	0.0007	>4.3
	13	105.2	>50	0.0004	>0.5
	15	637.1	>50	0.7016	>0.1
	16	239.4	377.4	0.1069	1.6

Note: ^1^ NDM = not determined. The mean inhibitory concentration (IC_50_) value was expressed as the mean of three independent experiments. Statistically significant differences (*p*-value) were determined between the means of SUM149PT and MCF-10A cells. F1 and F2 lack *p*-values since the viability curves running between both cell lines (SUM149PT [0–12.5 µM], MCF-10A [0–70 µM]) were different and it was impossible to perform the analysis.

## Data Availability

Data are contained within the article and Supplementary Materials.

## Supplementary Materials

The following supporting information can be downloaded at https://www.mdpi.com/article/10.3390/ph17070864/s1: Figure S1: Appearance of the native *G. multiplicatum*, and *G. martinicense*; Figure S2: Fractionated native *Ganoderma* spp. medicinal compounds; Figure S3: Cell viability assays of *G. multiplicatum* in SUM149PT (TNBC cell line) and MCF-10A (human noncancerous cell line); Figure S4: Cell viability assays of *G. martinicense* in SUM149PT (TNBC cell line) and MCF-10A (human noncancerous cell line); Figure S5: Compounds of *G. multiplicatum* with statistically significant differences (*p*-values); Figure S6: Compounds of *G. martinicense* with statistically significant differences (*p*-values).

## References

[1] Kinnel B., Singh S.K., Oprea-Ilies G., Singh R. (2023). Targeted therapy and mechanisms of drug resistance in breast cancer. Cancers.

[2] Almansour N.M. (2022). Triple-negative breast cancer: A brief review about epidemiology, risk factors, signaling pathways, treatment and role of artificial intelligence. Front. Mol. Biosci..

[3] Prakash O., Hossain F., Danos D., Lassak A., Scribner R., Miele L. (2020). Racial disparities in triple negative breast cancer: A review of the role of biologic and non-biologic factors. Front. Public Health.

[4] Zhang W., Bai Y., Sun C., Lv Z., Wang S. (2022). Racial and regional disparities of triple negative breast cancer incidence rates in the United States: An analysis of 2011-2019 NPCR and SEER incidence data. Front. Public Health.

[5] Yap Y. (2023). Outcomes in breast cancer—Does ethnicity matter?. ESMO Open.

[6] Rosario-Rosado R.V., Nazario C.M., Hernández-Santiago J., Schelske-Santos M., Mansilla-Rivera I., Ramírez-Marrero F.A., Ramos-Valencia G., Climent C., Nie J., Freudenheim J.L. (2020). Breast cancer in a Caribbean population in transition: Design and implementation of the atabey population-based case-control study of women in the San Juan metropolitan area in Puerto Rico. Int. J. Environ. Res. Public Health.

[7] Yin L., Duan J.-J., Bian X.-W., Yu S.-C. (2020). Triple-negative breast cancer molecular subtyping and treatment progress. Breast Cancer Res..

[8] Quirindongo-Rivera C., Rullán-Varela V., Underill Z., Rivera M., Ortiz-Ortiz K.J., Martínez-Montemayor M.M. (2022). Characteriza-tion of inflammatory breast cancer in Hispanic women from Puerto Rico. J. Cancer.

[9] Li Y., Zhang H., Merkher Y., Chen L., Liu N., Leonov S., Chen Y. (2022). Recent advances in therapeutic strategies for triple-negative breast cancer. J. Hematol. Oncol..

[10] Yao H., He G., Yan S., Chen C., Song L., Rosol T.J., Deng X. (2017). Triple-negative breast cancer: Is there a treatment on the horizon?. Oncotarget.

[11] Lin Z., Lin Z., Yang B. (2019). Ganoderma (Lingzhi) in Traditional Chinese Medicine and Chinese Culture. Ganoderma and Health.

[12] Wang L., Li J.-Q., Zhang J., Li Z.-M., Liu H.-G., Wang Y.-Z. (2020). Traditional uses, chemical components and pharmacological activities of the genus *Ganoderma* P. Karst.: A review. RSC Adv..

[13] Sharifi A., Naseri M.H., Jahedi S., Sarkary B., Rooz S.S.K., Khosravani S.M., Kalantar E. (2012). Antimicrobial potentials of crude fractions of polysaccharides of *Ganoderma* spp.. Afr. J. Microbiol. Res..

[14] Suárez-Arroyo I.J., Loperena-Alvarez Y., Rosario-Acevedo R., Martínez-Montemayor M.M. (2017). *Ganoderma* spp.: A promising adjuvant treatment for breast cancer. Medicines.

[15] Barbieri A., Quagliariello V., Del Vecchio V., Falco M., Luciano A., Amruthraj N.J., Nasti G., Ottaiano A., Berretta M., Iaffaioli R.V. (2017). Anticancer and anti-inflammatory properties of *Ganoderma lucidum* extract effects on melanoma and triple-negative breast cancer treatment. Nutrients.

[16] Merdivan S., Lindequist U. (2017). Ergosterol peroxide: A mushroom-derived compound with promising biological activities—A review. Int. J. Med. Mushrooms.

[17] Lu J., He R., Sun P., Zhang F., Linhardt R.J., Zhang A. (2020). Molecular mechanisms of bioactive polysaccharides from *Ganoderma* lucidum (Lingzhi), a review. Int. J. Biol. Macromol..

[18] Seweryn E., Ziała A., Gamian A. (2021). Health-promoting of polysaccharides extracted from *Ganoderma lucidum*. Nutrients.

[19] Zhou L., Chen H.-P., Li X., Liu J.-K. (2022). Ganoaustralins A and B, unusual aromatic triterpenes from the mushroom *Ganoderma australe*. Pharmaceuticals.

[20] Galappaththi M.C.A., Patabendige N.M., Premarathne B.M., Hapuarachchi K.K., Tibpromma S., Dai D.-Q., Suwannarach N., Rapior S., Karunarathna S.C. (2022). A review of *Ganoderma* triterpenoids and their bioactivities. Biomolecules.

[21] Rijia A., Krishnamoorthi R., Rasmi M., Mahalingam P.U., Kim K.-S. (2024). Comprehensive analysis of bioactive compounds in wild *Ganoderma applanatum* mushroom from Kerala, South India: Insights into dietary nutritional, mineral, antimicrobial, and antioxidant activities. Pharmaceuticals.

[22] Martínez-Montemayor M.M., Acevedo R.R., Otero-Franqui E., Cubano L.A., Dharmawardhane S.F. (2011). *Ganoderma* lucidum (reishi) inhibits cancer cell growth and expression of key molecules in inflammatory breast cancer. Nutr. Cancer.

[23] Suárez-Arroyo I.J., Rosario-Acevedo R., Aguilar-Perez A., Clemente P.L., Cubano L.A., Serrano J., Schneider R.J., Martínez-Montemayor M.M. (2013). Anti-tumor effects of *Ganoderma* lucidum (Reishi) in inflammatory breast cancer in in vivo and in vitro models. PLoS ONE.

[24] Suárez-Arroyo I.J., Rios-Fuller T.J., Feliz-Mosquea Y.R., Lacourt-Ventura M., Leal-Alviarez D.J., Maldonado-Martinez G., Cubano L.A., Martínez-Montemayor M.M. (2016). *Ganoderma lucidum* combined with the EGFR tyrosine kinase inhibitor, erlotinib synergize to reduce inflammatory breast cancer progression. J. Cancer.

[25] Rios-Fuller T.J., Ortiz-Soto G., Lacourt-Ventura M., Maldonado-Martinez G., Cubano L.A., Schneider R.J., Martinez-Montemayor M.M. (2018). *Ganoderma lucidum* extract (GLE) impairs breast cancer stem cells by targeting the STAT3 pathway. Oncotarget.

[26] Acevedo-Díaz A., Ortiz-Soto G., Suárez-Arroyo I.J., Zayas-Santiago A., Montemayor M.M.M. (2019). *Ganoderma lucidum* extract reduces the motility of breast cancer cells mediated by the RAC–lamellipodin axis. Nutrients.

[27] Ma Q., Zhang S., Yang L., Xie Q., Dai H., Yu Z., Zhao Y. (2022). Lanostane Triterpenoids and Ergostane Steroids from *Ganoderma luteomarginatum* and Their Cytotoxicity. Molecules.

[28] Min B.-S., Gao J.-J., Nakamura N., Hattori M. (2000). Triterpenes from the spores of *Ganoderma* lucidum and their cytotoxicity against meth-A and LLC tumor cells. Chem. Pharm. Bull..

[29] Qin F.-Y., Chen Y.-Y., Zhang J.-J., Cheng Y.-X. (2022). Meroterpenoid dimers from *Ganoderma* mushrooms and their biological activities against triple negative breast cancer cells. Front. Chem..

[30] Wu J.M., Hsieh T.-C. (2011). Suppression of proliferation and oxidative stress by extracts of *Ganoderma* lucidum in the ovarian cancer cell line OVCAR-3. Int. J. Mol. Med..

[31] Rai R.D., Singh S.K., Yadav M.C., Rai R.D., Singh S.K., Yadav M.C., Tewar R.P. (2007). Biological diversity in the genus *Ganoderma*. Mushroom Biology and Biotechnology.

[32] Campi M., Mancuello C.R., Ferreira F.P., Maubet Y., Cristaldo E., Robledo G. (2021). Bioactive compounds and antioxidant activity of four native species of the *Ganoderma*taceae Family (Agaricomycetes) from Paraguay. Int. J. Med. Mushrooms.

[33] Loyd A.L., Linder E.R., Smith M.E., Blanchette R.A., Smith J.A. (2019). Cultural characterization and chlamydospore function of the *Ganoderma*taceae present in the Eastern United States. Mycologia.

[34] Campi M., Mancuello C., Ferreira F., Maubet Y., Cristaldo E., Gayoso E., Robledo G. (2023). Does the source matter? Phenolic compounds and antioxidant activity from mycelium in liquid medium, wild and cultivated fruiting bodies of the neotropical species *Ganoderma* tuberculosum. J. Microbiol. Biotechnol. Food Sci..

[35] Tu Y., Jeffries C., Ruan H., Nelson C., Smithson D., Shelat A.A., Brown K.M., Li X.-C., Hester J.P., Smillie T. (2010). Automated high-throughput system to fractionate plant natural products for drug discovery. J. Nat. Prod..

[36] Loyd A.L., Barnes C.W., Held B.W., Schink M.J., Smith M.E., Smith J.A., Blanchette R.A. (2018). Elucidating “lucidum”: Distinguishing the diverse laccate *Ganoderma* species of the United States. PLoS ONE.

[37] Hay R., American Type Culture Collection (1992). ATCC Quality Control Methods for Cell Lines.

[38] Foulkes W.D., Smith I.E., Reis-Filho J.S. (2010). Triple-negative breast cancer. N. Engl. J. Med..

[39] Li S., Fang Y. (2021). Research on the mechanism of pulsatilla potentially useful for the treatment of triple negative breast cancer based on network pharmacology. Res. Sq..

[40] Komatsu M., Yoshimaru T., Matsuo T., Kiyotani K., Miyoshi Y., Tanahashi T., Rokutan K., Yamaguchi R., Saito A., Imoto S. (2012). Molecular features of triple negative breast cancer cells by genome-wide gene expression profiling analysis. Int. J. Oncol..

[41] Robles A.J., Du L., Cichewicz R.H., Mooberry S.L. (2016). Maximiscin induces DNA damage, activates DNA damage response pathways, and has selective cytotoxic activity against a subtype of triple-negative breast cancer. J. Nat. Prod..

[42] Bhattacharya T., Dutta S., Akter R., Rahman H., Karthika C., Nagaswarupa H.P., Murthy H.C.A., Fratila O., Brata R., Bungau S. (2021). Role of Phytonutrients in Nutrigenetics and Nutrigenomics Perspective in Curing Breast Cancer. Biomolecules.

[43] Chiou Y., Li S., Ho C., Pan M. (2018). Prevention of Breast Cancer by Natural Phytochemicals: Focusing on Molecular Targets and Combinational Strategy. Mol. Nutr. Food Res..

[44] Israel B.B., Tilghman S.L., Parker-Lemieux K., Payton-Stewart F. (2018). Phytochemicals: Current strategies for treating breast cancer (review). Oncol. Lett..

[45] Rizeq B., Gupta I., Ilesanmi J., AlSafran M., Rahman M., Ouhtit A. (2019). The power of phytochemicals combination in cancer chemoprevention. J. Cancer.

[46] Vini R., Sreeja S. (2015). *Punica granatum* and its therapeutic implications on breast carcinogenesis: A review. BioFactors.

[47] Braakhuis A.J., Campion P., Bishop K.S. (2016). Reducing Breast Cancer Recurrence: The Role of Dietary Polyphenolics. Nutrients.

[48] Forcados G.E., James D.B., Sallau A.B., Muhammad A., Mabeta P. (2017). Oxidative Stress and Carcinogenesis: Potential of Phytochemicals in Breast Cancer Therapy. Nutr. Cancer.

[49] Jeitler M., Michalsen A., Frings D., Hübner M., Fischer M., Koppold-Liebscher D.A., Murthy V., Kessler C.S. (2020). Significance of medicinal mushrooms in integrative oncology: A narrative review. Front. Pharmacol..

[50] Wang M., Yu F. (2022). Research progress on the anticancer activities and mechanisms of polysaccharides from *Ganoderma*. Front. Pharmacol..

[51] Boh B. (2013). *Ganoderma* lucidum: A potential for biotechnological production of anti-cancer and immunomodulatory drugs. Recent Pat. Anti-Cancer Drug Discov..

[52] Zhao S., Ye G., Fu G., Cheng J.-X., Yang B.B., Peng C. (2011). *Ganoderma* lucidum exerts anti-tumor effects on ovarian cancer cells and enhances their sensitivity to cisplatin. Int. J. Oncol..

[53] Shin M.-J., Chae H.-J., Lee J.W., Koo M.H., Kim H.-J., Seo J.B., Yanillia S., Park S.H., Lo H.E., Kim S.-H. (2022). Lucidumol A, purified directly from *Ganoderma* lucidum, exhibits anticancer effect and cellular inflammatory response in colorectal cancer. Evid. Based Complement. Altern. Med..

[54] Andrejč D.C., Knez Ž., Marevci M.K. (2022). Antioxidant, antibacterial, antitumor, antifungal, antiviral, anti-inflammatory, and nevro-protective activity of *Ganoderma* lucidum: An overview. Front. Pharmacol..

[55] Bhosle S., Ranadive K., Bapat G., Garad S., Deshpande G., Vaidya J. (2010). Taxonomy and Diversity of *Ganoderma* from the Western parts of Maharashtra (India). Mycosphere.

[56] Correia de Lima Júnior N., Baptista Gibertoni T., Malosso E. (2014). Delimitación de algunos *Ganoderma* (*Ganoderma*taceae) lacados neotropicales: Filogenia molecular y morfología. Rev. Biol. Trop..

[57] Morera G., Lupo S., Alaniz S., Robledo G. (2021). Diversity of the *Ganoderma* species in Uruguay. Neotrop. Biodivers..

[58] Nguyen T.T.T., Nguyen T.T.T., Nguyen H.D., Nguyen T.K., Pham P.T.V., Tran L.T.T., Tran M.H. (2023). Integrating in silico and in vitro studies to screen anti-*Staphylococcus aureus* activity from Vietnamese *Ganoderma multiplicatum* and *Ganoderma sinense*. Nat. Prod. Commun..

[59] Zhou L.-W., Nakasone K.K., Burdsall H.H., Ginns J., Vlasák J., Miettinen O., Spirin V., Niemelä T., Yuan H.-S., He S.-H. (2016). Polypore diversity in North America with an annotated checklist. Mycol. Prog..

