# Supplementary material for: Selective Antineoplastic Potential of Fractionated Caribbean Native Ganoderma Species Extracts on Triple-Negative Breast Cancer Cells
Source: Pharmaceuticals (Basel). 2024 Jul 1;17(7):864. doi: 10.3390/ph17070864 (PMC11279663; doi:10.3390/ph17070864)
Supplement: Supplementary file 1 [file pharmaceuticals-17-00864-s001.zip › pharmaceuticals-3073097-supplementary.pdf]

Supplementary Files

Selective Anti-Neoplastic Potential of Fractionated Caribbean Native *Ganoderma* species Extracts on Triple-Negative Breast Cancer Cells

I.	**Additional experimental procedures**	**Page**
	a) Appearance of the native *G. multiplicatum*, and *G. martinicense*	2
	b) Fractionated native *Ganoderma* spp. medicinal compounds	3
	c) Supporting cell viability assay graphs (*G. multiplicatum*)	4-5
	d) Supporting cell viability assay graphs (*G. martinicense*)	6-7
	e) Supporting statistically significant different (*p* value) graphs (*G. multiplicatum*)	8-9
	f) Supporting statistically significant different (*p* value) graphs (*G. martinicense*)	10-11

Data Availability Statement

The original contributions presented in the study are included in the article/supplementary material, further inquiries can be directed to the corresponding author/s.

a) Appearance of the native *G. multiplicatum*, and *G. martinicense*

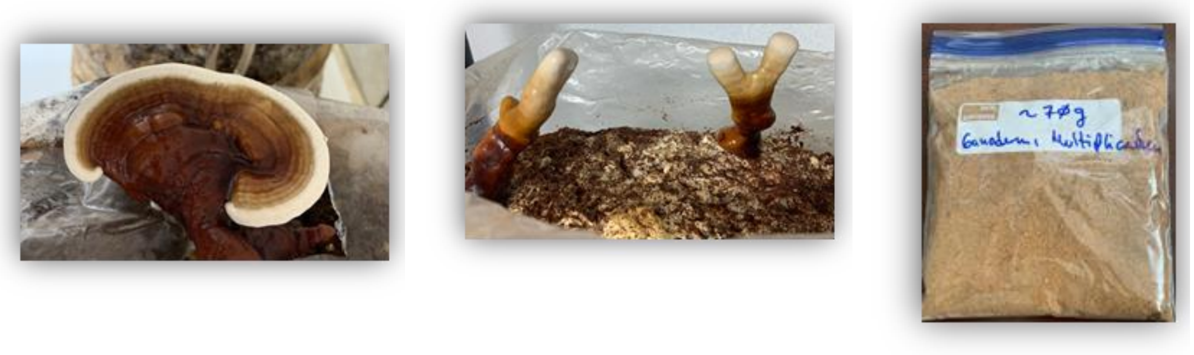

(a) **(b)** **(c)**

Figure S1. Appearance of the native *Ganoderma multiplicatum*, and *Ganoderma martinicense*. Photos of the harvested medicinal mushroom: (a) *G. multiplicatum*, and (b) *G. martinicense*; and the samples dried and pulverized: (c) *G. multiplicatum*. (Photos were provided by: Huerto Rico, LLC).

b) Fractionated native *Ganoderma* spp. medicinal compounds

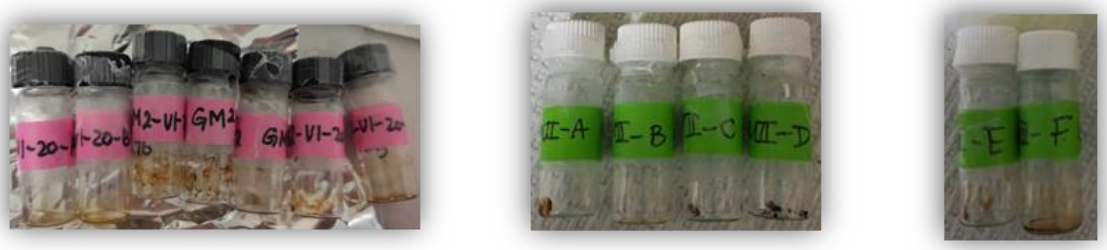

(a) (b)

Figure S2. Fractionated native *Ganoderma* spp. medicinal compounds: (a) *G. multiplicatum* (pink label), and (b) *G. multiplicatum* (green label) fractions for reconstitution.

c) Supporting cell viability assay graphs (*G. multiplicatum*)

i

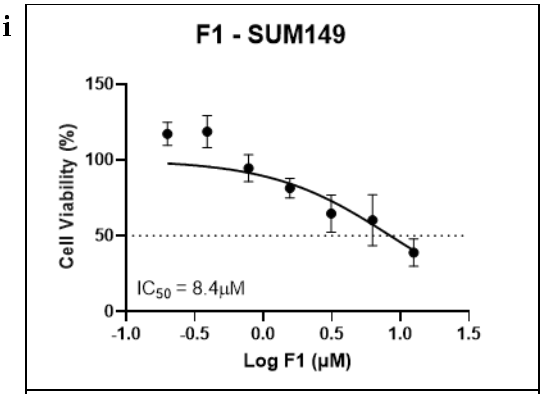

ii

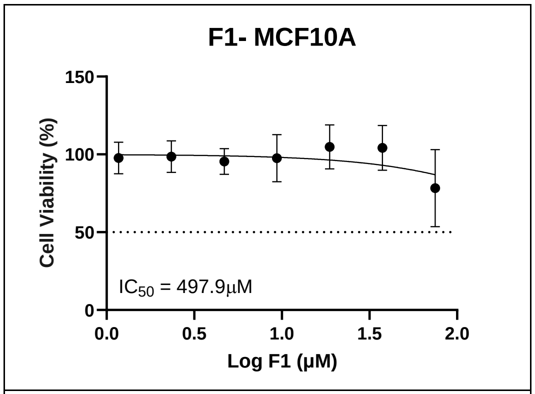

iii

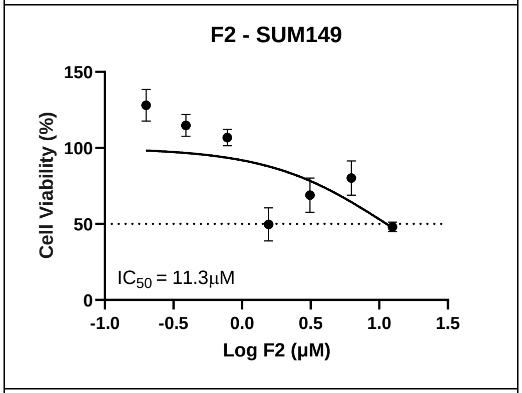

iv

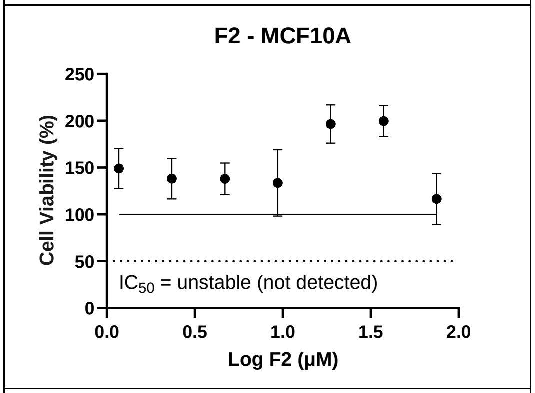

v

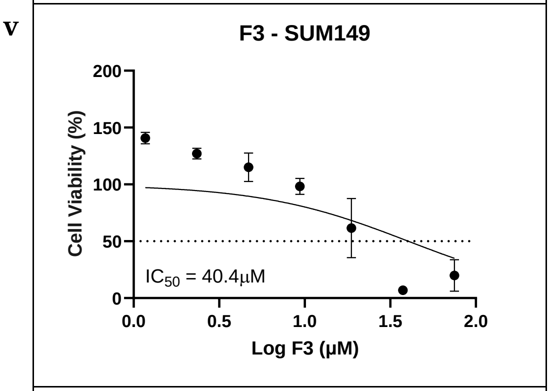

vi

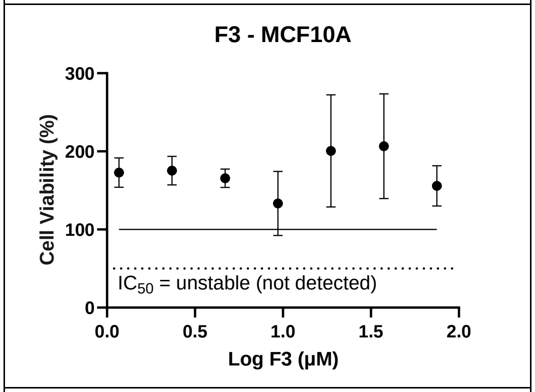

vii

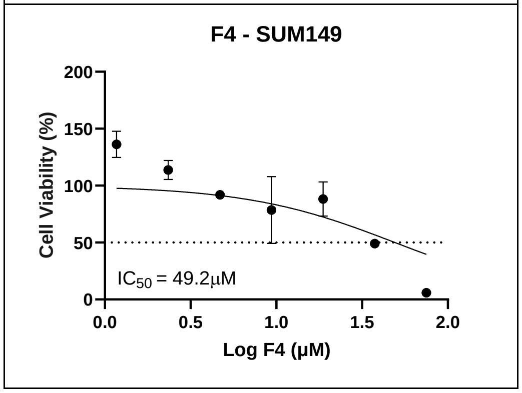

viii

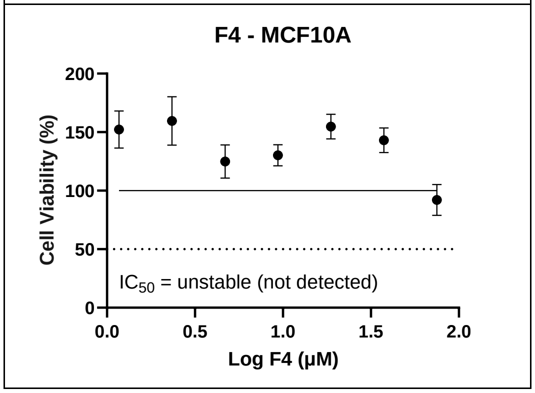

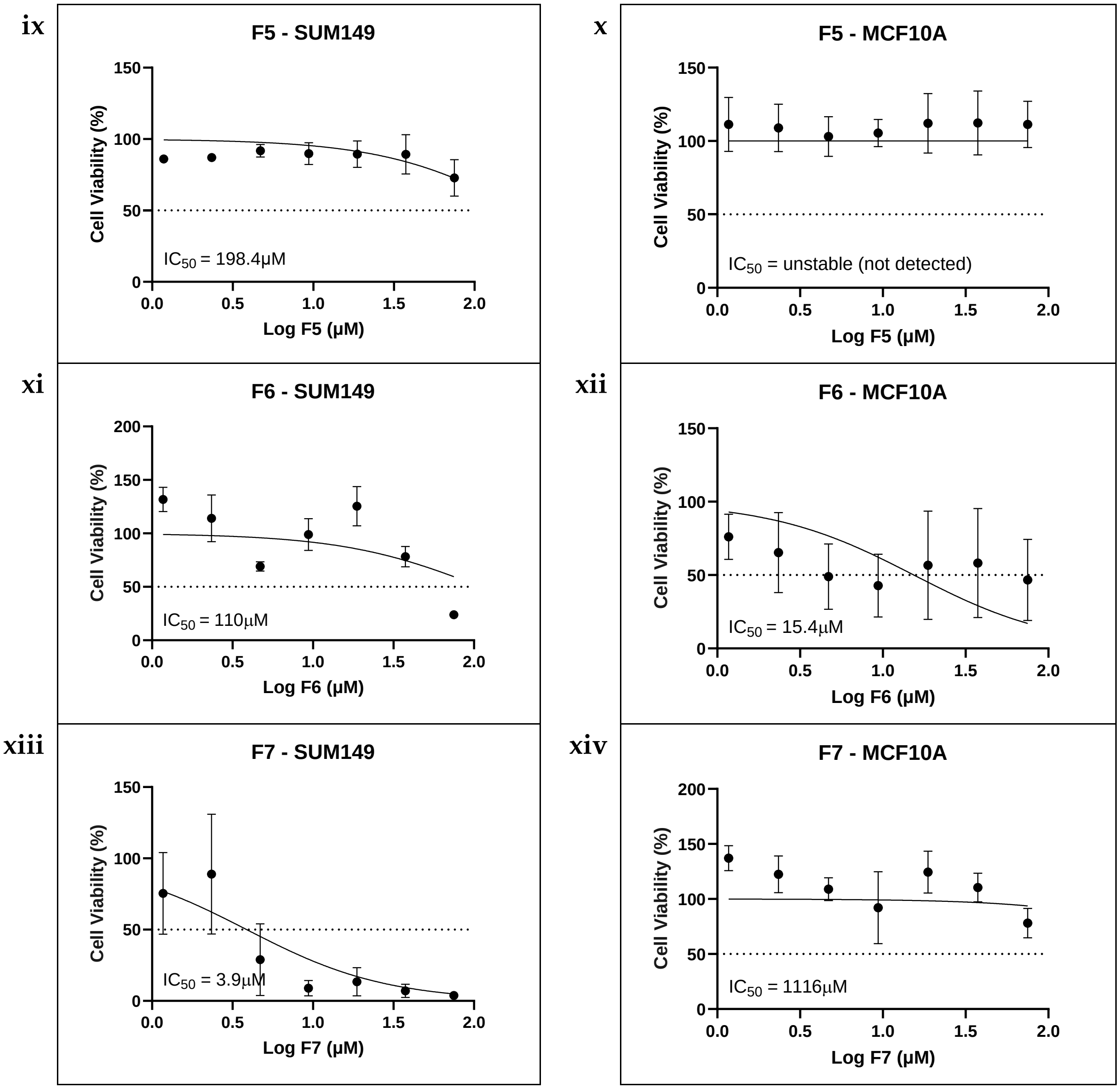

Figure S3. Cell viability assays of *Ganoderma multiplicatum* in SUM149PT (TNBC cell line) and MCF-10A (human non-cancerous cell line). The mean inhibitory concentrations (IC_{50}) value expressed as the mean of three independent experiments.

d) Supporting cell viability assay graphs (*G. martinicense*)

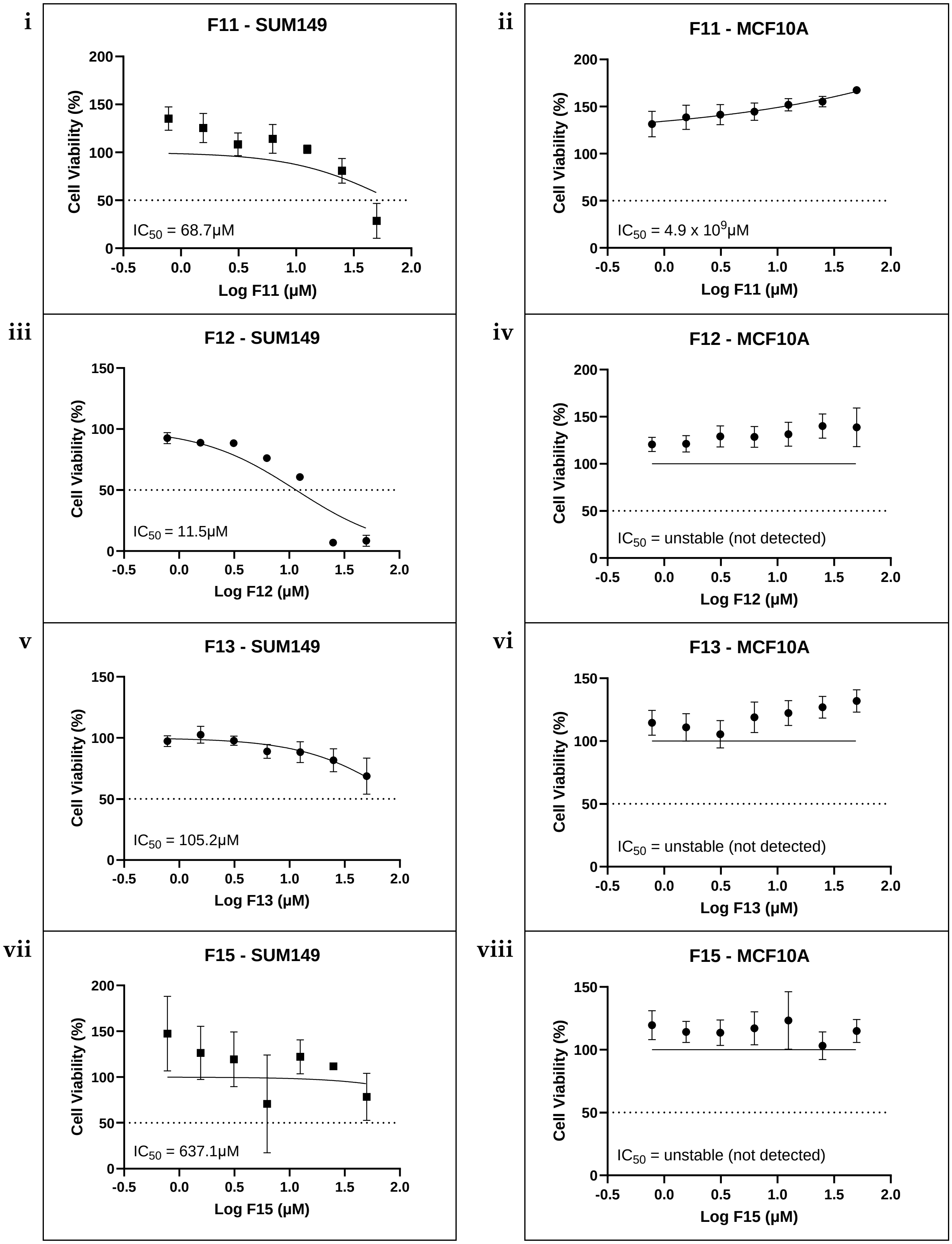

ix x

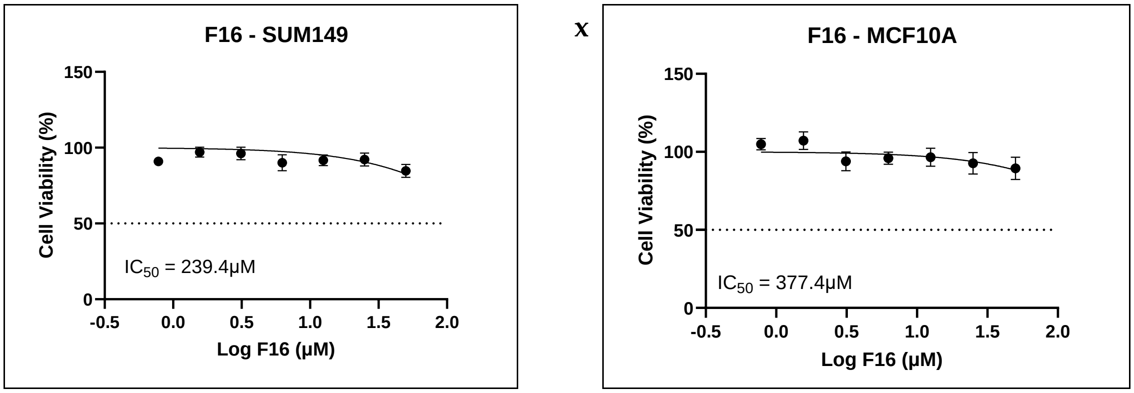

Figure S4. Cell viability assays of *Ganoderma martinicense* in SUM149PT (TNBC cell line) and MCF-10A (human non-cancerous cell line). The mean inhibitory concentrations (IC_{50}) value expressed as the mean of three independent experiments. *G. martinicense* fraction #14 did not cause any toxicity on either the cancer or the non-cancerous cells evaluated.

e) Supporting significant different (*P value*) graphs (*G. multiplicatum*)

i

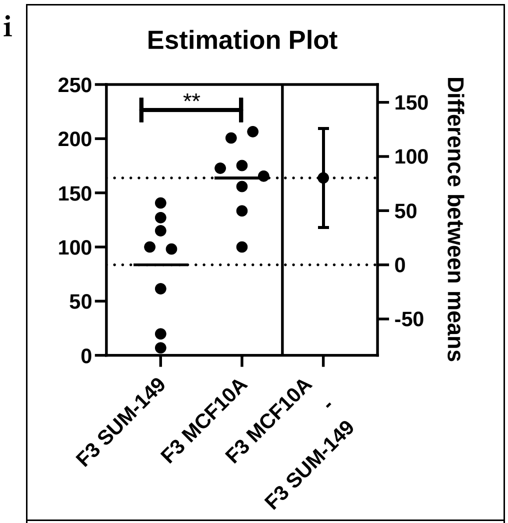

ii

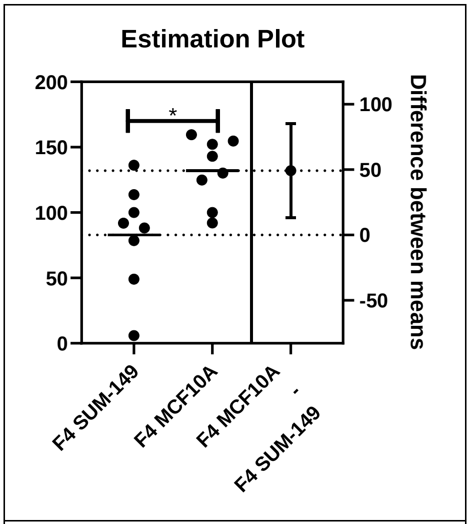

iii

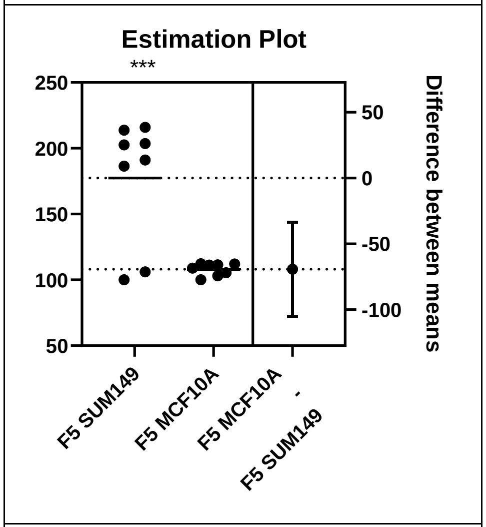

iv

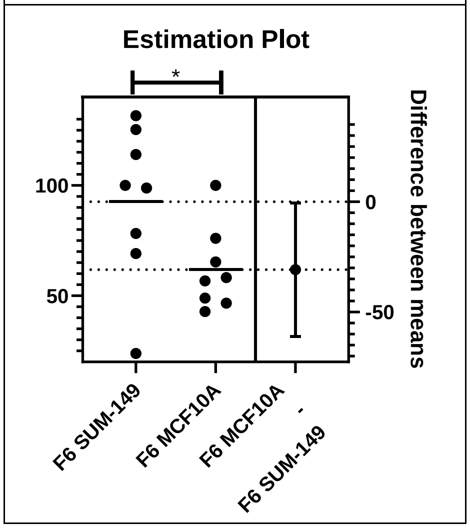

v

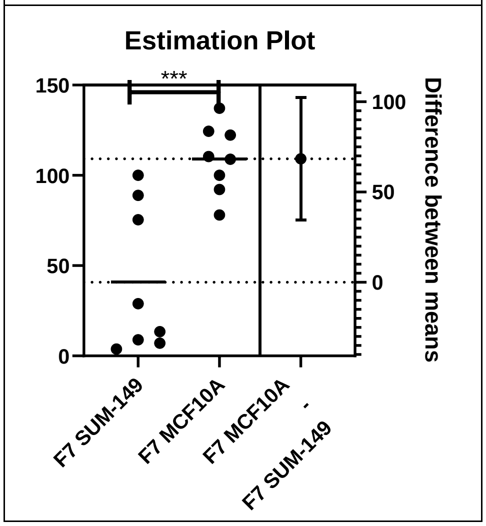

Figure S5. Compounds statistically significant different (*p value*) of *Ganoderma multiplicatum*. *P values* were determined between SUM149PT and MCF-10A cells means (* $p < 0.05$, ** $p < 0.01$, and *** $p < 0.001$). F1 and F2 lack a *p value* since the viability curves obtained between both cell lines (SUM149PT [0 - 12.5 μM], MCF10A [0 - 70 μM]) were different and it was not possible to perform the statistical analysis. Bars represent mean ± SEM of at least 3 biological replicates.

f) Supporting significant different (*P value*) graphs (*G. martinicense*)

i

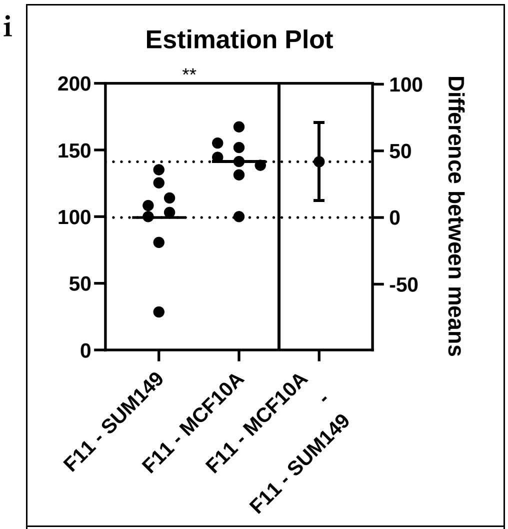

ii

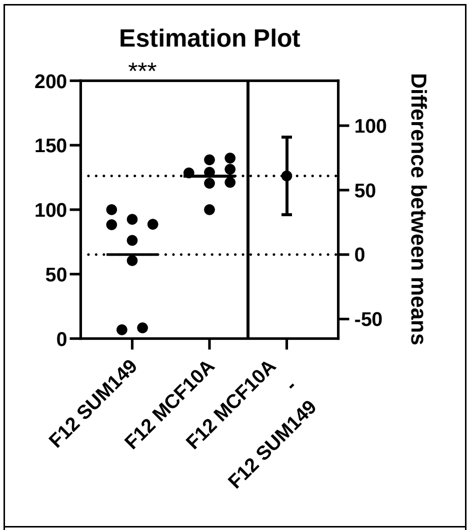

iii

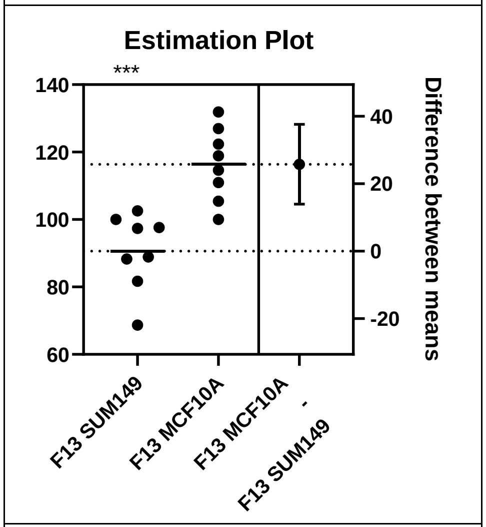

iv

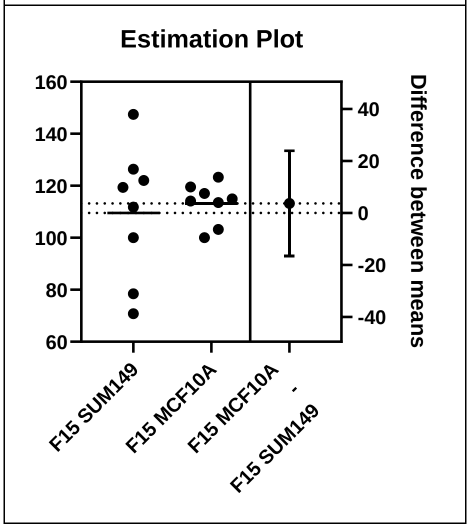

v

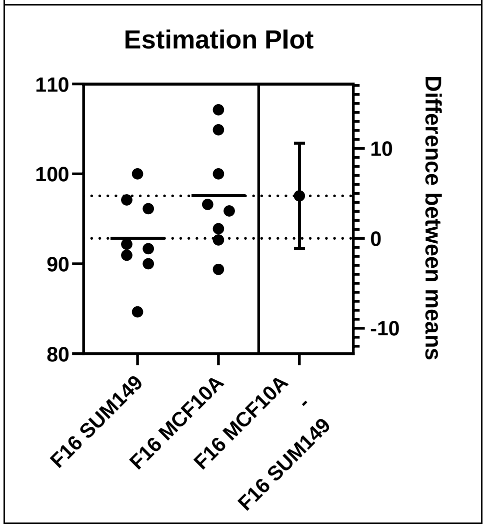

Figure S6. Compounds statistically significant different (*p value*) of *Ganoderma martinicense. P values* were determined between SUM149PT and MCF-10A cells means (** $p < 0.01$, and *** $p < 0.001$). Bars represent mean ± SEM of at least 3 biological replicates. *G. martinicense* F14 did not cause any toxicity on either the cancer or the non-cancerous cells evaluated.
